# Association Between Low Chinese Visceral Adiposity Index Values and Chronic Lung Disease in Older Adults: Cohort Study

**DOI:** 10.2196/78627

**Published:** 2026-05-20

**Authors:** Xiaorong Zhu, Hui Tian, Zhi Liu, Zhoubin Huang, Miaomiao Yuan, Zhangshun Tu, Kaiwang Cui, Jianping Liu, Xiangwen Gong

**Affiliations:** 1 Department of Respiratory and Critical Care Medicine, Ganzhou Key Laboratory of Respiratory Diseases, Ganzhou Institute of Respiratory Diseases, The Fifth People's Hospital of Ganzhou, Dongjiangyuan Road 666, Zhanggong District, Ganzhou, China, 86 0797-8528520; 2 Xingguo County Pulmonary Hospital, Ganzhou, China; 3 Gannan Medical University, Ganzhou, China; 4 Jiangxi Province Key Laboratory of Preventive Medicine, School of Public Health, Nanchang University, Nanchang, China

**Keywords:** chronic lung diseases, Chinese visceral adiposity index, China Health and Retirement Longitudinal Study, restricted cubic spline models, cohort study

## Abstract

**Background:**

Visceral fat content, a key indicator of obesity, has been increasingly linked to chronic lung diseases (CLDs). However, the precise relationship between the Chinese visceral adiposity index (CVAI) and CLD remains unclear.

**Objective:**

This study aimed to investigate the association between the CVAI and CLD risk.

**Methods:**

Data from 6695 participants aged ≥45 years in the China Health and Retirement Longitudinal Study were analyzed. Restricted cubic spline models were applied to evaluate dose-response relationships between the CVAI and CLD across 3 population subgroups. Cox models were used to assess the hazard ratios and 95% CIs for the relationship between CVAI quintiles and CLD incidence in the significantly associated population.

**Results:**

Restricted cubic spline analysis revealed a significant L-shaped relationship between the CVAI and CLD incidence in the older adult population (≥60 years; *P* value for overall=.01; *P* value for nonlinearity=.04), rather than in the middle-aged and older adult population (≥45 years; *P* value for overall=.12) or middle-aged population (45-59 years; *P* value for overall=.55). The inflection point of the CVAI for CLD risk was identified at 126 in the older adults. After multivariable adjustment, compared with participants in the fourth quintile (Q4), those in the first quintile (Q1) exhibited a significantly increased risk of CLD (hazard ratio=1.38, 95% CI 1.05‐1.83). The subgroup analyses, which were stratified by sociodemographic factors and lifestyle, showed consistent results across most subgroups (*P* value for interaction>.05).

**Conclusions:**

In adults aged ≥60 years, a lower CVAI was associated with a higher risk of CLD. No such association was observed in middle-aged adults.

## Introduction

Chronic lung diseases (CLDs), which are recognized as a critical global public health challenge [1], demonstrate a pathological spectrum encompassing diverse respiratory disorders, including chronic bronchitis, emphysema, cor pulmonale, and asthma. Epidemiological evidence indicates a high prevalence of these conditions among middle-aged and older adult populations, with substantial impacts on patients’ quality of life and considerable socioeconomic burdens. Current research has established tobacco smoke exposure, environmental pollution, and genetic predisposition as primary etiological factors [2], which collectively induce irreversible pulmonary functional impairment through sustained disruption of alveolar architecture and pulmonary tissue homeostasis.

In recent years, the interplay between metabolic dysregulation and chronic diseases has emerged as a pivotal research focus. Notably, aberrant visceral adiposity has been conclusively linked to type 2 diabetes mellitus, cardiovascular diseases, and metabolic syndrome [3,4]. While BMI remains widely used for obesity assessment, it fails to delineate the spatial distribution characteristics of adipose tissue [5]. The weight-adjusted waist circumference index, calculated as waist circumference divided by the square root of body weight, improves abdominal adiposity evaluation but retains limitations in clinical utility due to its regional specificity [6]. In contrast, the Chinese visceral adiposity index (CVAI) integrates biochemical parameters such as triglycerides and high-density lipoprotein cholesterol (HDL-C) to establish a multidimensional metabolic profiling system. Emerging evidence demonstrates the CVAI’s robust prognostic associations with hypertension [7], chronic kidney disease, metabolic syndrome [8], and diabetes mellitus [9], solidifying its role as a critical biomarker for quantifying visceral fat–related metabolic derangements.

Emerging evidence suggests that visceral adiposity may impair pulmonary function through mechanisms involving proinflammatory cytokine release and exacerbated oxidative stress [10]. Nevertheless, the relationship between the CVAI and chronic pulmonary diseases remains underexplored. Leveraging the large-scale dataset from the China Health and Retirement Longitudinal Study (CHARLS), this study aims to elucidate the epidemiological association between the CVAI and chronic pulmonary diseases, thereby providing mechanistic insights to inform the development of metabolism-targeted prevention and therapeutic strategies for respiratory disorders.

## Methods

### Ethical Considerations

The CHARLS survey was reviewed and approved by the institutional review board of Peking University (IRB00001052-11015). Before participation, all respondents were provided with a comprehensive introduction to the potential risks and benefits of the study. Thereafter, each participant provided written informed consent and repository consent, permitting the storage and secondary sharing of their data for research purposes. Participants’ privacy and confidentiality were strictly protected: personal identifiers were removed, and data were stored on secure servers accessible only to authorized study personnel. Data sharing was conducted in accordance with applicable data protection regulations. Participants did not receive direct financial compensation; however, they were informed about the potential indirect benefits of participation, such as contributing to national health research and policy development.

### Study Design and Study Population

The data for this study were obtained from the CHARLS, which was a national representative study. The details of study design and assessment protocols for the CHARLS have been clearly detailed in a previous study [11]. In summary, the CHARLS is the first high-quality microdatabase designed to accurately represent families and individuals aged ≥45 years in China. This database serves as a vital resource for examining the challenges associated with population aging in China. Between 2011 and 2012, a sample of over 17,000 individuals aged ≥45 years was recruited from 450 communities and administrative villages across 28 provinces nationwide, using a multistage probability sampling method. Since the initial recruitment, these participants have undergone biennial follow-up assessments.

Data from waves 1 to 5 (collected in 2011, 2013, 2015, 2017, and 2020, respectively) were included in this study. At baseline (wave 1), 17,596 participants were enrolled. Participants were excluded based on the following criteria: (1) age <45 years, (2) missing information on the CVAI, (3) missing covariate data, and (4) lost to follow-up during waves 2-5.

The start time was defined as the date of the 2011 CHARLS baseline interview. Event time was calculated in years from baseline to the earliest of either the first self-reported CLD or the wave 5 (2020) interview. Censoring was applied to participants who were lost to follow-up at any point during the follow-up period (waves 2‐5) or remained free of CLD at study end.

### Assessment of the CVAI

The CVAI is a reliable and applicable index for evaluation of visceral fat dysfunction in Chinese, which has been used in many studies. It was calculated with sex-specific formulas as follows [12]:

Females: CVAI = −187.32 + 1.71 × age + 4.23 × BMI + 1.12 × WC + 39.76 × Log_10_TG − 11.66 × HDL-C

Males: CVAI = −267.93 + 0.68 × age + 0.03 × BMI + 4.00 × WC + 22.00 × Log_10_TG − 16.32 × HDL-C

### Assessment of CLD

CLD was assessed by asking participants whether they have been diagnosed with CLD by a doctor. They were classified as patients with CLD if the answer was positive. In contrast, they were not classified as patients with CLD if the answer was negative.

### Assessment of Covariates

Covariates include the following: demographic data (age, sex, education, area of residence, and marital status), lifestyles (smoking and alcohol consumption), and chronic disease. Age was divided into 2 categories: middle-aged (45‐59 years) and older (≥60 years). Education was categorized into 3 groups: illiterate, primary school and below, or middle school and above. Residence was grouped into urban or village. Marital status was divided into 2 categories: married and others (including unmarried, separated, divorced, and widowed). Smoking and alcohol consumption were categorized into 3 groups: never, former, and current. Chronic diseases were assessed in a similar manner to CLD and grouped into yes or no categories.

### Statistical Analysis

Boruta is an all-relevant feature selection wrapper algorithm that identifies which features are important and which are not. We used it to determine the important features associated with incident CLD [13].

Restricted cubic spline (RCS) analyses were performed with the “rcssci” R package (version 0.4.0; R Foundation for Statistical Computing) [14] to assess the dose-response associations of the CVAI with CLD in three populations: (1) middle-aged and older adults (≥45 years), (2) middle-aged adults (45‐59 years), and (3) older adults (≥60 years). A 2-piecewise Cox model was applied when a cutoff point was identified in the RCS analysis. The number of knots was automatically selected by the “rcssci” R package through minimization of the Akaike information criterion. Three RCS models ultimately used 3 knots placed at the 10th, 50th, and 90th percentiles, ensuring a parsimonious yet flexible fit to any nonlinear relationship.

Subsequently, participants exhibiting a significant association between the CVAI and CLD were grouped by CVAI quintiles. Differences in CVAI quintile groups were tested using 1-way ANOVA for normally distributed data, the Kruskal-Wallis test for nonnormally distributed data, and the *χ*^2^ test for categorical data. Cox models were used to assess the hazard ratios (HRs) and 95% CIs for the relationship between CVAI quintiles and CLD incidence. Model 1 was the crude model. In model 2, we adjusted for age, sex, education, marital status, residence, smoking, and alcohol consumption. In model 3, we further adjusted for several chronic diseases (hypertension, dyslipidemia, diabetes, heart attack, stroke, stomach disease, and memory-related disease). The proportional hazards assumptions for all Cox models were assessed using Schoenfeld residuals and were not violated (all *P* values >.05).

Finally, subgroup analyses stratified by sex (male or female), education (illiterate or literate), marital status (married or other), residence (village or urban), smoking status (no or yes), and alcohol consumption (no or yes) were conducted. Additionally, the interactions of the CVAI with the stratified factors were tested using the likelihood ratio test by comparing models with and without the interaction term.

The Stata 15 SE (StataCorp) and R (version 4.4.1; R Foundation for Statistical Computing) were used for analyses, and statistical significance was defined as 2-tailed *P* values <.05. *P* values reported throughout the study are defined as follows: *P* value for overall indicates the overall significance of RCS model, *P* value for nonlinearity assesses whether the association exhibits a non-linear relationship in RCS analyses, and *P* value for interaction evaluates the significance of interaction terms between covariates and the main exposure.

## Results

### Study Population

**Figure 1.**
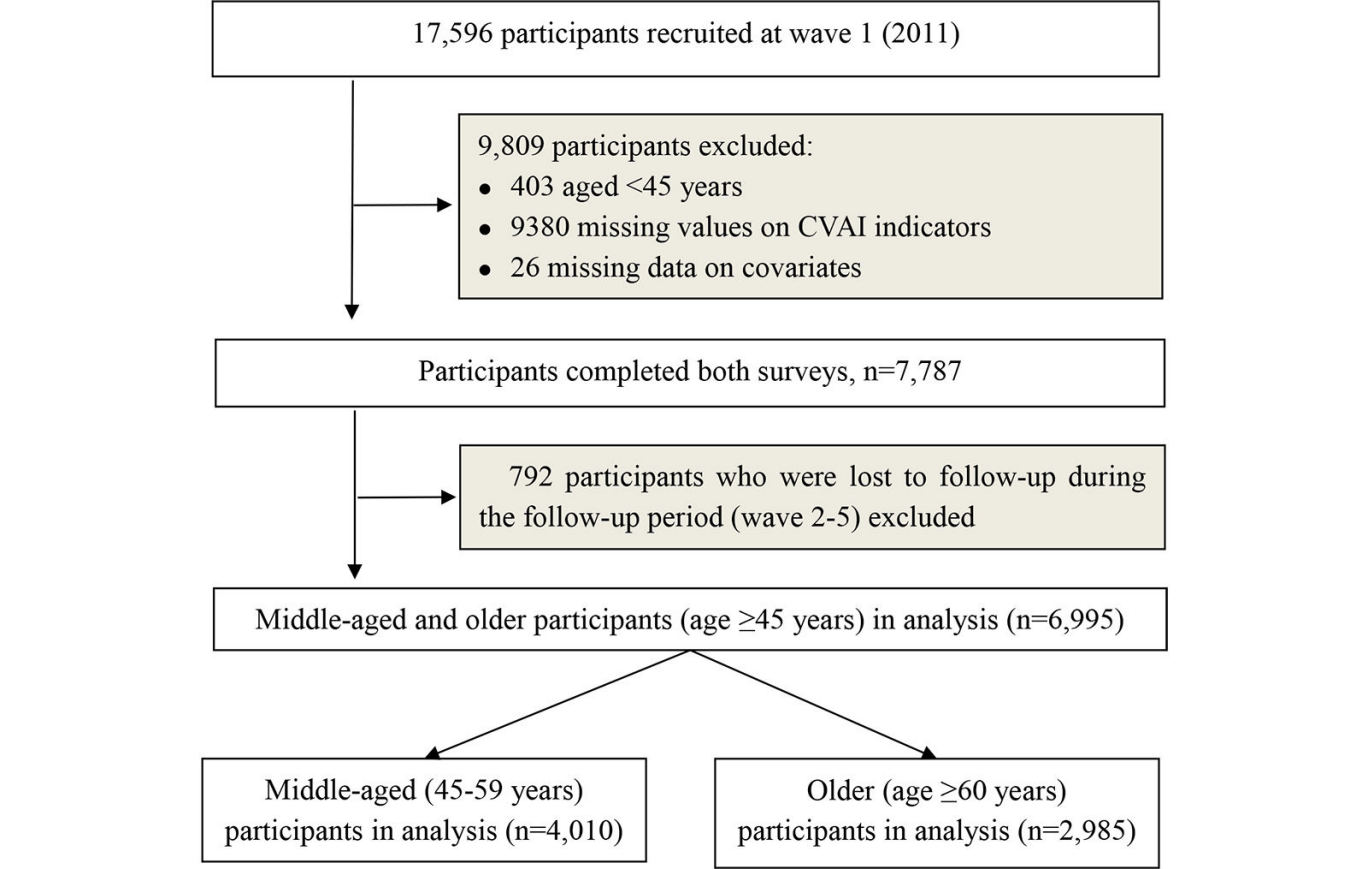
Flowchart of study participants. CVAI: Chinese visceral adiposity index.

A total of 6695 participants aged ≥45 years with complete data were included in the analysis. Of these, 4010 were middle-aged (45–59 years old) and 2685 were older (≥60 years old). The process of participant selection, including all exclusions at each step, is illustrated in Figure 1.

### Feature Selection

**Figure 2.**
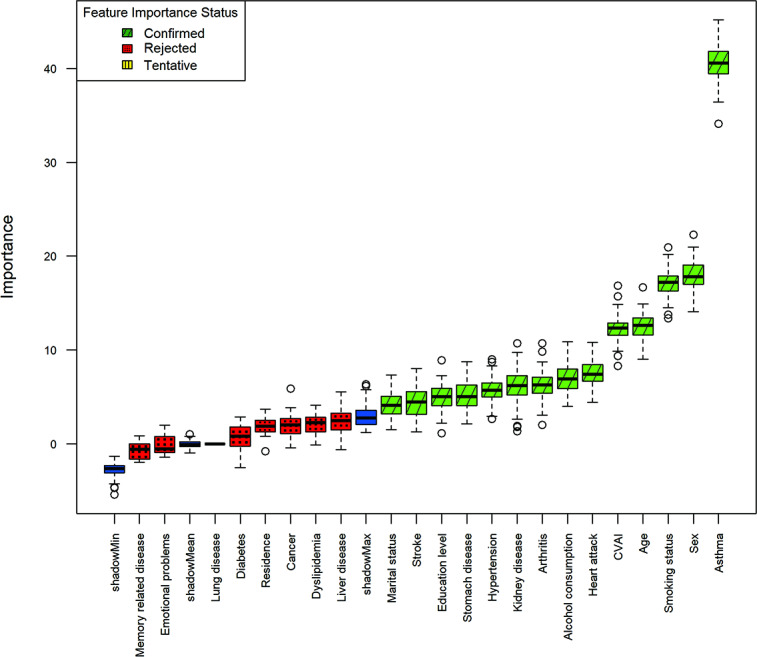
Feature selection for chronic lung disease incidence using the Boruta algorithm (ie, Boruta feature importance). ShadowMin, shadowMax, and shadowMean indicate the minimum, maximum, and mean importance values of shadow (randomized) features used internally by the Boruta algorithm. CVAI: Chinese visceral adiposity index.

Figure 2 shows 14 features strongly associated with incident CLD identified by the Boruta algorithm. The 14 confirmed variables are the CVAI, age, sex, education level, marital status, smoking status, alcohol consumption, hypertension, heart attack, stroke, kidney disease, stomach disease, arthritis, and asthma. Residence, though falling below the shadow-feature threshold, was retained on the basis of prior evidence and clinical experience [15,16].

### Dose-Response Relationship

**Figure 3.**
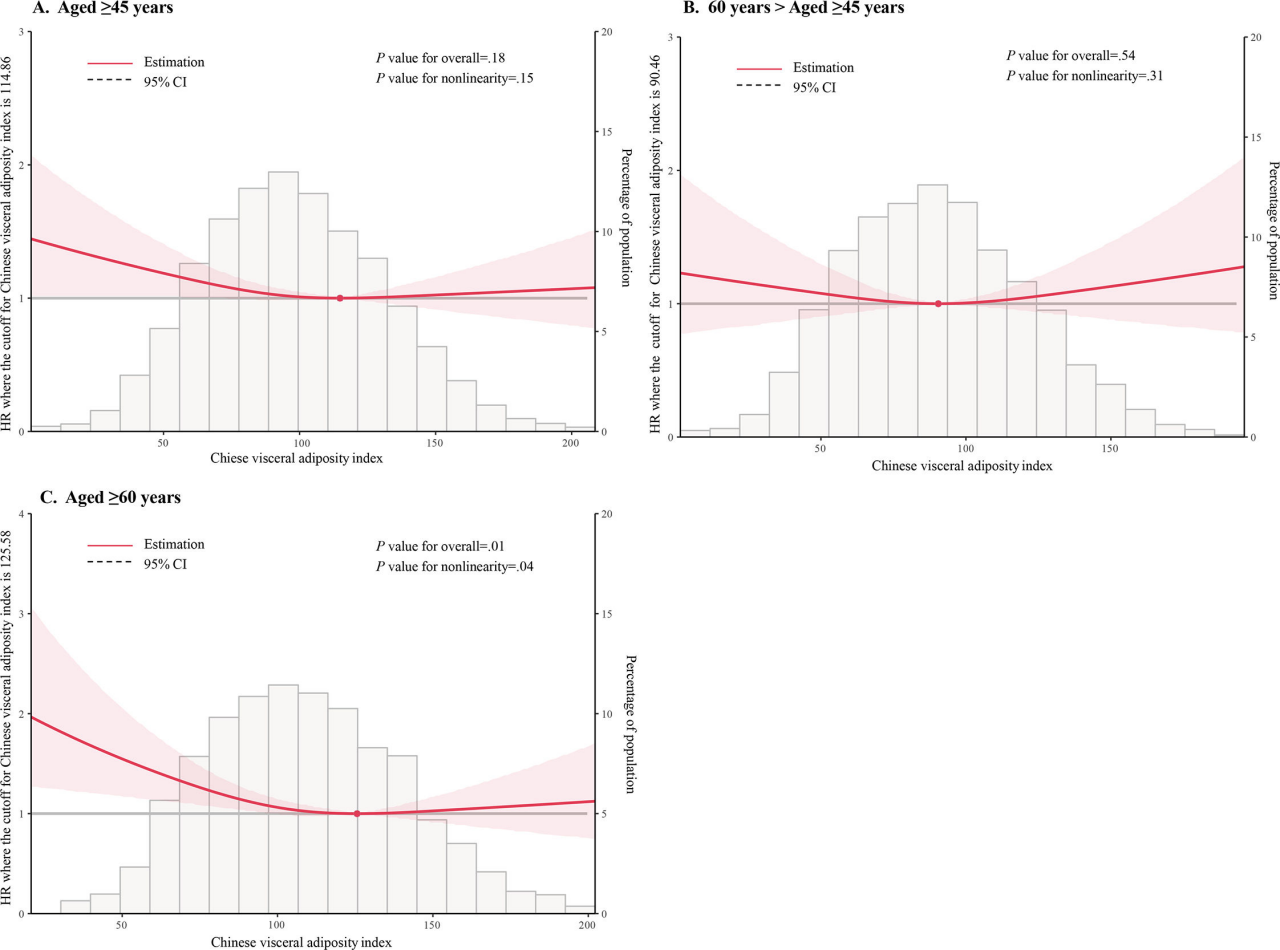
Dose-response relationship between the Chinese visceral adiposity index (CVAI) and chronic lung disease. Spline analyses were adjusted for age, sex, education, marital status, residence, smoking, alcohol consumption, hypertension, heart attack, stroke, kidney disease, stomach disease, arthritis, and asthma. HR: hazard ratio.

Figure 3 shows the results of the RCS analyses examining the association between the CVAI and CLD across different age groups. The results suggested a significant L-shaped relationship between the CVAI and CLD in older adults (aged ≥60 years; *P* value for overall=.01; *P* value for nonlinearity=.04). The cutoff point for the CVAI was 126. In a multiadjusted 2-piecewise Cox model, when the CVAI was below 126, each 1-point decrease in the CVAI was associated with a 0.6 % increase in CLD risk (HR=1.006, 95% CI 1.002-1.011; *P*=.007). When the CVAI was above 126, CLD risk showed a nonsignificant increasing trend with each 1-point increase in the CVAI (HR=1.001, 95% CI 0.992-1.010; *P*=.86).

However, the CVAI was not associated with CLD in either the middle-aged and older adult population (aged ≥45 years; *P* value for overall=.12) or the middle-aged population (aged 45‐59 years; *P* value for overall=.55).

### Baseline Characteristics of the Older Adult Population

According to the L-shaped association between the CVAI and CLD in the older adult population, the CVAI was categorized in quintiles: Q1 (CVAI<80), Q2 (80≤CVAI<98), Q3 (98≤CVAI<116), Q4 (116≤CVAI<136), and Q5 (136≤CVAI). Table 1 presents the characteristics of the older adult population. Participants with a higher CVAI were more likely to be older, female, married, living in urban areas, non-smokers, and non-drinkers, and to have lower educational attainment, hypertension, a history of heart attack, stroke, and stomach disease.

**Table 1.** Characteristics of older adult participants (aged ≥60 years) by quintiles of the Chinese visceral adiposity index (N=2985).

Characteristics	Chinese visceral adiposity index^a^					*P* value
	Q1 (n=597)	Q2 (n=597)	Q3 (n=597)	Q4 (n=597)	Q5 (n=597)	
Age (years), mean (SD)	64.75 (4.51)	66.39 (5.34)	66.95 (5.87)	67.30 (5.89)	68.38 (6.36)	<.001
Sex, n (%)						<.001
Male	332 (55.61)	340 (56.95)	277 (46.40)	259 (43.38)	239 (40.03)	
Female	265 (44.39)	257 (43.05)	320 (53.60)	338 (56.62)	358 (59.97)	
Education level, n (%)						<.001
Illiterate	199 (33.33)	201 (33.67)	233 (39.03)	210 (35.18)	241 (40.37)	
Primary school and below	302 (50.59)	308 (51.59)	260 (43.55)	276 (46.23)	231 (38.69)	
Middle school and above	96 (16.08)	88 (14.74)	104 (17.42)	111 (18.59)	125 (20.94)	
Marital status, n (%)						.008
Married	88 (14.74)	98 (16.42)	111 (18.59)	127 (21.27)	128 (21.44)	
Other	509 (85.26)	499 (83.58)	486 (81.41)	470 (78.73)	469 (78.56)	
Residence, n (%)						<.001
Village	467 (78.22)	439 (73.53)	403 (67.50)	365 (61.14)	331 (55.44)	
Urban	130 (21.78)	158 (26.47)	194 (32.50)	232 (38.86)	266 (44.56)	
Smoking status, n (%)						<.001
Never smoker	291 (48.74)	305 (51.09)	371 (62.14)	376 (62.98)	398 (66.67)	
Former smoker	49 (8.21)	56 (9.38)	55 (9.21)	59 (9.88)	73 (12.23)	
Current smoker	257 (43.05)	236 (39.53)	171 (28.64)	162 (27.14)	126 (21.11)	
Alcohol consumption, n (%)						<.001
Never drinker	311 (52.09)	316 (52.93)	347 (58.12)	365 (61.14)	391 (65.49)	
Former drinker	49 (8.21)	62 (10.39)	63 (10.55)	57 (9.55)	63 (10.55)	
Current drinker	237 (39.70)	219 (36.68)	187 (31.32)	175 (29.31)	143 (23.95)	
Hypertension, n (%)	84 (14.07)	123 (20.60)	168 (28.14)	209 (35.01)	313 (52.43)	<.001
Heart attack, n (%)	48 (8.05)	54 (9.05)	73 (12.23)	92 (15.41)	124 (20.77)	<.001
Stroke, n (%)	14 (2.35)	4 (0.67)	14 (2.35)	16 (2.68)	31 (5.19)	<.001
Kidney disease, n (%)	21 (3.52)	23 (3.85)	29 (4.86)	31 (5.19)	34 (5.70)	.34
Stomach disease, n (%)	164 (27.47)	135 (22.61)	142 (23.79)	123 (20.60)	108 (18.09)	.002
Arthritis, n (%)	222 (37.25)	218 (36.52)	206 (34.51)	211 (35.34)	223 (37.35)	.82
Asthma, n (%)	8 (1.34)	11 (1.84)	12 (2.01)	12 (2.01)	18 (3.02)	.35

a Q1-Q5 represent the quintiles of Chinese Visceral Adiposity Index (CVAI) in the older population: Q1 (lowest quintile, CVAI<80), Q2 (80≤CVAI<98), Q3 (98≤CVAI<116), Q4 (116≤CVAI<136), and Q5 (highest quintile, CVAI≥136).

### CVAI and CLD in the Older Adult Population

During follow-up (mean 8.42, SD 1.73 years; total 25,132 person-years), 527 participants developed incident CLD, yielding an incidence density of 20.97 per 1000 person-years.

The association of the CVAI and CLD in the older adult population is shown in Table 2. We selected Q4 as the reference group because the cutoff point (126) falls within the range of the Q4 group (116≤CVAI<136). After adjusting multiple covariates, the HR of participants in the Q1 group was 1.38 (95% CI 1.05-1.83) compared to the Q4 group. However, no significant difference was observed between the Q4 group and either the Q2, Q3, and Q5 groups. Kaplan-Meier plots by CVAI quintiles also showed the lowest CVAI group to have the highest CLD incident risk (Multimedia Appendix 1).

**Table 2.** Hazard ratios (HRs) and 95% CIs for the association between the Chinese visceral adiposity index (CVAI) and chronic lung disease in older adults (N=2985).^a^

CVAI	Cases, n (%)	Model 1^b^		Model 2^c^		Model 3^d^	
		HR (95% CI)	*P* value	HR (95% CI)	*P* value	HR (95% CI)	*P* value
Q1	127 (21.27)	1.42 (1.04-1.77)	.01	1.35 (1.03-1.78)	.03	1.38 (1.05-1.83)	.02
Q2	104 (17.42)	1.16 (0.87-1.54)	.30	1.11 (0.84-1.48)	.45	1.14 (0.86-1.52)	.36
Q3	103 (17.25)	1.13 (0.85-1.50)	.39	1.13 (0.85-1.50)	.40	1.13 (0.85-1.49)	.40
Q4	91 (15.24)	1 (reference)	—^e^	1 (reference)	—	1 (reference)	—
Q5	102 (17.09)	1.11 (0.84-1.47)	.46	1.14 (0.86-1.51)	.37	1.07 (0.80-1.42)	.65

a Q1-Q5 represent the quintiles of CVAI in the older population: Q1 (lowest quintile, CVAI<80), Q2 (80≤CVAI<98), Q3 (98≤CVAI<116), Q4 (116≤CVAI<136), and Q5 (highest quintile, CVAI≥136).

b Model 1 was unadjusted.

c Model 2 was adjusted for age, sex, education, marital status, residence, smoking, and alcohol consumption.

d Model 3 was adjusted for age, sex, education, marital status, residence, smoking, alcohol consumption, hypertension, heart attack, stroke, kidney disease, stomach disease, arthritis, and asthma.

e Not applicable.

### Subgroup Analysis

Subgroup analyses stratified by sociodemographic factors and lifestyle were performed after full adjustment (Table 3). The results showed that the relationship between the CVAI and CLD was consistent across most subgroups (*P* value for interaction>.05). A significant interaction for education and the CVAI was observed (*P* value for interaction=.03). Among the illiterate older adult population, compared with the Q4 group, the HRs were 2.03 (1.21-3.46) and 1.70 (1.07-2.76) in the Q1 and Q5 groups, respectively. Conversely, the CVAI was not related with CLD in the literate older adult population.

**Table 3.** The subgroup analysis for the association between the Chinese visceral adiposity index (CVAI) and chronic lung disease in older adults (N=2984).

Subgroup	CVAI^a^^,^^b^					*P* value for interaction
	Q1, HR^c^ (95% CI)	Q2, HR (95% CI)	Q3, HR (95% CI)	Q4, HR (95% CI)	Q5, HR (95% CI)	
Sex						.83
Male	1.28 (0.87-1.88)	1.09 (0.74-1.59)	1.10 (0.74-1.63)	1 (reference)	0.87 (0.57-1.34)	
Female	1.52 (0.98-2.30)	1.26 (0.82-1.93)	1.20 (0.80-1.81)	1 (reference)	1.26 (0.85-1.86)	
Education						.03
Illiterate	2.03 (1.21-3.46)	1.16 (0.87-2.18)	1.72 (1.04-2.87)	1 (reference)	1.70 (1.07-2.76)	
literate	1.13 (0.81-1.58)	1.12 (0.80-1.56)	0.90 (0.63-1.28)	1 (reference)	0.84 (0.59-1.21)	
Marital status						.25
Married	1.35 (0.72-2.55)	1.13 (0.59-2.17)	1.09 (0.53-1.86)	1 (reference)	1.56 (0.88-2.73)	
Other	1.38 (0.99-1.89)	1.14 (0.83-1.57)	1.17 (0.85-1.60)	1 (reference)	0.91 (0.65-1.28)	
Residence						.94
Village	1.43 (1.02-2.00)	1.12 (0.79-1.59)	1.15 (0.81-1.63)	1 (reference)	1.11 (0.77-1.61)	
Urban	1.31 (0.77-2.22)	1.24 (0.75-2.07)	1.17 (0.72-1.90)	1 (reference)	1.03 (0.62-1.55)	
Smoking status						.98
No	1.28 (0.89-1.85)	1.09 (0.76-1.58)	1.14 (0.81-1.62)	1 (reference)	1.09 (0.78-1.54)	
Yes	1.68 (1.06-2.66)	1.31 (0.81-2.10)	1.17 (0.71-1.93)	1 (reference)	1.04 (0.61-1.78)	
Alcohol consumption						.52
No	1.50 (1.06-2.13)	1.17 (0.81-1.67)	1.03 (0.72-1.47)	1 (reference)	1.11 (0.79-1.57)	
Yes	1.20 (0.74-1.93)	1.06 (0.66-1.72)	1.29 (0.80-2.08)	1 (reference)	0.91 (0.53-1.57)	

a Model was adjusted for age, sex, education, marital status, residence, smoking, alcohol consumption, hypertension, heart attack, stroke, kidney disease, stomach disease, arthritis, and asthma.

b Q1-Q5 represent the quintiles of CVAI in the older population: Q1 (lowest quintile, CVAI<80), Q2 (80≤CVAI<98), Q3 (98≤CVAI<116), Q4 (116≤CVAI<136), and Q5 (highest quintile, CVAI≥136).

c HR: hazard ratio.

## Discussion

### Principal Findings

This study systematically investigated the relationship between the CVAI and CLD in a cohort from the CHARLS. The results demonstrated a significant L-shaped relationship between the CVAI and CLD, with a low CVAI being negatively correlated with the risk of CLDs. This finding not only improves the understanding of the association between visceral fat and respiratory diseases but also provides a quantifiable biomarker reference value for the risk stratification of CLD based on body fat distribution in clinical practice.

In the study, we revealed that participants with lower visceral adiposity exhibited an elevated risk of chronic pulmonary diseases, particularly pronounced in older adult populations. In cardiovascular research, the functional heterogeneity of adipose tissue across anatomical depots contributes to differential cardiovascular risks, with visceral fat being mechanistically implicated in metabolic syndrome and cardiovascular disease pathogenesis through multiple pathways [17]. Consequently, clinical obesity metrics have evolved beyond weight-centric indices such as BMI to incorporate visceral adiposity surrogates. The CVAI—integrating BMI, waist circumference, triglycerides, and HDL-C—has been validated as a robust predictor of diabetes, cardiovascular disease, hypertension, and metabolic syndrome in Chinese cohorts [18-21]. However, no prior studies have explored the CVAI–chronic pulmonary disease nexus. Intriguingly, a study using Asia-Pacific BMI criteria demonstrated an inverse correlation between BMI and lung function [22], whereas analyses applying World Health Organization (WHO) BMI thresholds revealed U-shaped associations for forced expiratory volume in one second and diffusing capacity for carbon monoxide. This discrepancy underscores the critical influence of region-specific obesity classification criteria on pulmonary function assessments. Our CVAI-based analysis identified an L-shaped relationship between visceral adiposity and chronic pulmonary disease incidence in Chinese adults. While existing literature predominantly reports linear inverse correlations between obesity severity and lung function decline [23,24], most studies contrast pulmonary metrics between overweight or obese and normal-weight groups [25,26]. Notably, the current evidence base overlooks pulmonary dysfunction risks associated with hypoadiposity. Clinically, malnutrition-induced hypoadiposity warrants attention, as it impairs respiratory muscle architecture and contractility, heightening fatigue susceptibility and ventilatory dysfunction [27-30]. Concurrent protein deficiency compromises immunoglobulin and complement production, exacerbating immunosuppression and infection susceptibility, thereby aggravating respiratory impairment in underweight populations [29-31]. These mechanisms may underlie the observed association between a low CVAI and heightened chronic pulmonary disease risk.

Excessive visceral adiposity has been implicated in pulmonary function impairment and elevated chronic pulmonary disease incidence. While our findings suggested an increasing trend in CLD risk when the CVAI was above 126, this trend was not statistically significant (HR=1.001, 95% CI 0.992-1.010, *P*=.86). Nevertheless, the obesity–lung function relationship remains multifaceted. Most studies corroborate an inverse association between obesity and pulmonary function, though paradoxically, some report attenuated lung function decline—for example, slower forced expiratory volume in one second reduction—in obese individuals [32]. Notably, overweight or obesity has demonstrated a protective effect against mortality in patients with chronic obstructive pulmonary disease [33], aligning with the “obesity paradox,” wherein excess adiposity correlates with improved clinical outcomes in chronic disease populations [34]. Adiposity may serve as a nutritional reserve, particularly critical for older, frail, or comorbid patients. Overweight or obese patients with chronic obstructive pulmonary disease exhibit higher fat-free mass and respiratory muscle mass compared to underweight or normal-weight counterparts—a physiological advantage in compensating for elevated airway resistance and airflow obstruction [35]. These factors may explain the survival benefits observed in obese patients with chronic respiratory conditions [36]. Thus, the CVAI–chronic pulmonary disease association warrants further investigation, particularly through disease-specific analyses or site-specific assessments of visceral fat distribution. Future studies should disentangle the dualistic effects of adiposity-metabolic detriment versus cardiopulmonary reserve to reconcile these seemingly contradictory epidemiological observations.

In the subgroup analysis, higher educational attainment appeared to buffer the effect of an unfavorable CVAI on CLD risk. The reason may be that individuals with higher education usually possess better health literacy and self-management abilities, enabling them to offset the negative effects of unreasonable visceral adipose tissue through active lifestyles and regular health check-ups.

Inevitably, there are some limitations in our study. First, data on chronic pulmonary disease were collected via self-reported questionnaires, introducing potential recall bias. Older adults are more likely to forget previous diagnoses, and CLD is a condition with a low diagnosis rate in China. Thus, the true association between a low CVAI and CLD may be underestimated. Future studies should link electronic medical records, conduct pulmonary function tests, or perform chest imaging verification to reduce recall bias. Second, some other confounders, including physical activity, diet quality, or detailed socioeconomic position, were not adjusted for. In theory, higher levels of these factors could attenuate the association between the CVAI and CLD. Finally, the study population was restricted to individuals aged >45 years, limiting generalizability to younger adult demographics.

### Conclusions

In conclusion, this study is the first to demonstrate an L-shaped association between the CVAI and CLD in the older adult population, with a low CVAI linked to a higher risk of CLD, but not in middle-aged individuals. These findings, while preliminary, highlight the need for validation through large-scale, multicenter prospective studies to elucidate age-stratified mechanisms linking visceral adiposity dynamics to respiratory health outcomes.

## Supplementary material

10.2196/78627 Multimedia Appendix 1 Kaplan-Meier plots.

## Abbreviations

CHARLS: China Health and Retirement Longitudinal Study
CLD: chronic lung disease
CVAI: Chinese visceral adiposity index
HDL-C: high-density lipoprotein cholesterol
HR: hazard ratio
RCS: restricted cubic spline
WHO: World Health Organization
