# Supplementary figures and images for: Association Between Low Chinese Visceral Adiposity Index Values and Chronic Lung Disease in Older Adults: Cohort Study
Source: JMIR Public Health Surveill. 2026 May 20;12:e78627. doi: 10.2196/78627 (PMC13189530; doi:10.2196/78627)

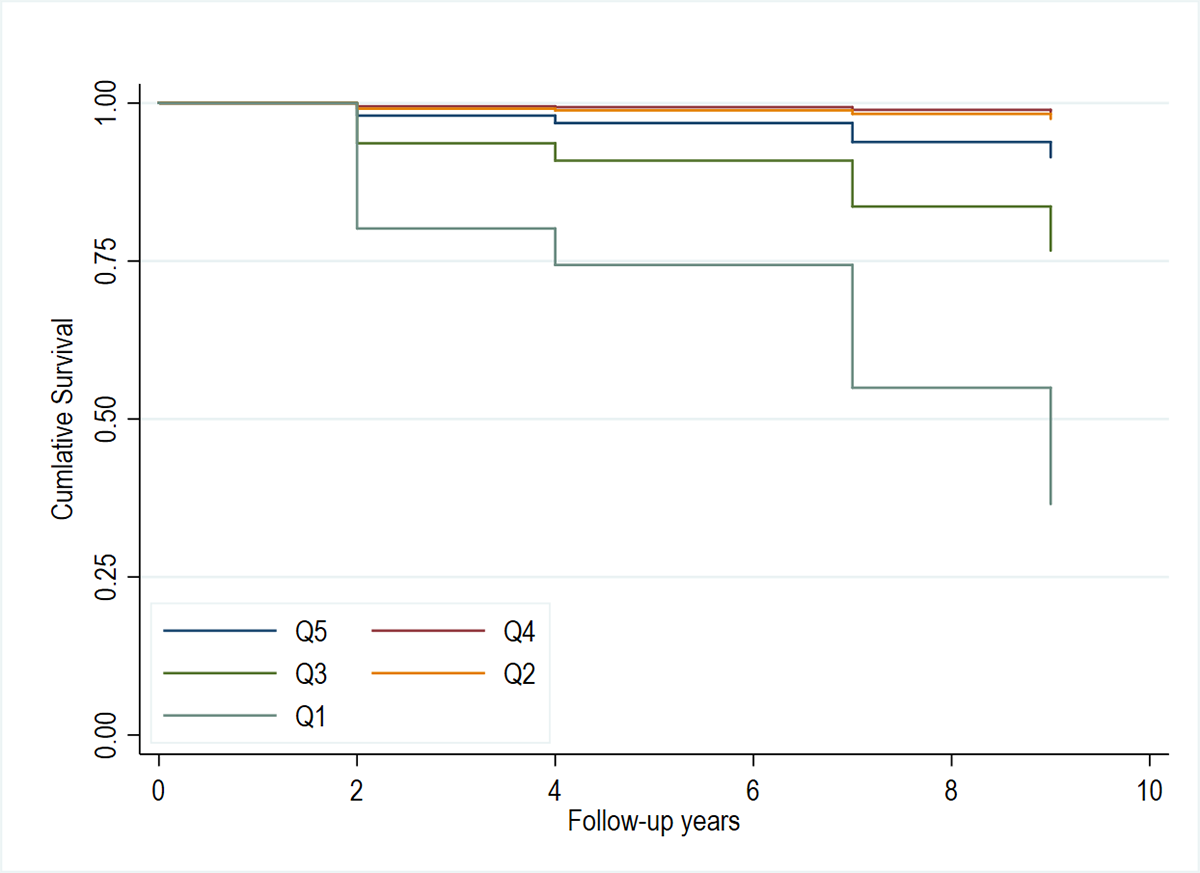

Supplement: Multimedia Appendix 1 [file publichealth-v12-e78627-s001.png]
